# The Effectiveness of Routine Cerclage in In Vitro Fertilization (IVF) Twins

**DOI:** 10.7759/cureus.65328

**Published:** 2024-07-25

**Authors:** Meena Samant, Divya Suman, Ummul Hasanat Tasneem, Ritu Singh, Poonam Lal

**Affiliations:** 1 Obstetrics and Gynecology, Kurji Holy Family Hospital, Patna, IND; 2 Obstetrics and Gynecology, Vaidyam Multispeciality Hospital, Patna, IND; 3 Obstetrics and Gynecology, Rajshree Medical College, Patna, IND; 4 Obstetrics and Gynecology, All India Institute of Medical Sciences, Patna, Patna, IND

**Keywords:** ivf pregnancies, pregnancy, cerclage, assisted reproductive technology (art), maternofetal outcome, macdonalds cerclage, pregnancy outcome, twins, ivf, prophylactic cervical cerclage

## Abstract

Introduction: The incidence of twin pregnancies is on the rise worldwide due to assisted reproductive technologies. Cervical dilatation is a frequent complication and can be considered a cause of premature births in twin pregnancies. In vitro fertilization (IVF) twins are more prone to preterm delivery. Routine cervical cerclage with normal cervical length is not recommended in twins, but studies were not done on IVF twins. So, this study aims to evaluate the effectiveness of routine transvaginal cerclage in twin IVF pregnancies with normal cervical length on maternofetal outcome.

Materials and methods: A retrospective case-control study was conducted at a maternity hospital in eastern India from January 2016 to December 2019 with over 21800 deliveries. Two groups were taken, cases were those IVF twins with normal cervical length in whom cervical cerclage was done as they have referred to our hospital from the IVF centers for cerclage and in control no-cervical cerclage as they are referred from other IVF centers where cerclage was not a routine protocol. We have excluded cerclage done for history, ultrasound indicated, uterine anomalies, and monochorionic twins. Fifteen participants were recruited in both cases and controls.

Results: In our study 2 (6.66%), 4 (13%), and 26 (86%) were IVF twins delivered at a gestational age of <28 weeks, 28-32 weeks, and 32-37 weeks, respectively. The mean age of participants, mean gestation age at delivery, and birth weight in cases and control were 35.27 ± 5.98 years (min: 23; max: 45), 32.40 ± 5.54 years (min: 25; max: 44); 34 weeks 2 days ± 3.28 (min: 31; max: 37), 33 weeks 5 days ± 1.66 (min: 25; max: 37); 1961.33 ± 340 gram, 1899.33 ± 437.48 gram, respectively with no statistical significant difference (p = 0.186, p = 1, p = 0.668, respectively)

Conclusion: Routine transvaginal cerclage is not effective in twin IVF pregnancies with normal cervical length for preventing preterm births. IVF twin women usually present at a late age and their birth weight are also low.

## Introduction

The incidence of twin pregnancies is on the rise worldwide due to assisted reproductive technologies (ART) [1]. In the United States of America (USA), the rate of twin births has increased by 76%, while that of triplets and higher orders is more than 400% [1].

At all times, twin pregnancies are considered high-risk due to the raised rate of maternal and fetal complications [2]. A high risk of fetal complications is mostly due to preterm delivery and low birth weight. According to the European Society on Infertility, the risk of extremely preterm birth (<28 weeks) has increased in twin in vitro fertilization (IVF) [3]. Furthermore, women who go for IVF are of increased age, which is a risk factor for complications. However, apart from maternal factors, there is a higher risk of preterm deliveries in dizygotic twin IVF women than in spontaneous dizygotic twins [4].

A higher incidence of cervical dilatation is a frequent complication and can be considered a cause of premature births in twin pregnancies [5]. So, routine prophylactic cerclage can be regarded as a prenatal intervention to prolong gestational age in twin pregnancies.

At present, the conclusions of international research on the prevention of preterm birth in twin pregnancies with cervical cerclage remain inconsistent. The guidelines developed by the International Federation of Gynecology and Obstetrics (FIGO) in 2021 recommended that for twins, the advantage seems more likely at shorter cervical lengths (<15 mm) [6]. However, there are studies on IVF twins that recommend prophylactic cervical cerclage [7-9] with the argument also that we have to do cervical cerclage in twin pregnancy with IVF for three reasons. The first reason for preterm labor is to prolong pregnancy so that corticosteroid therapy can be given to them [10]. The second reason is that it will prevent emergency cervical cerclage, which has no value as such [11,12]. The third reason is that it will minimize bed rest for the patients [9]. Several recent studies do not favor elective cervical cerclage [13-15], but these studies were not done in IVF pregnancies. So, this study aims to evaluate the effectiveness of prophylactic transvaginal cerclage in twin IVF pregnancies with normal cervical length on maternal and fetal outcomes.

## Materials and methods

Study design

A retrospective case-control study was conducted at a maternity hospital between January 2016 and December 2019 from a medical record paper. IVF-twin pregnant women were included in the study as cases who had prophylactic cerclage and controls were without cerclage. Inclusion criteria were cerclage in IVF twin pregnant women with normal cervical measurement ≥4 cm; twins after embryo reduction of a third embryo; or vanishing third twin also. Exclusion criteria were cerclage in ultrasound-indicated cases, history-indicated cerclage, cerclage in uterine anomalies, and monochorionic-monoamniotic twins.

Study procedure

The study was conducted at a hospital located in eastern India, which caters to maternity referral patients from IVF centers for cervical cerclage who do not have maternity facilities. Those IVF centers have a protocol to do cervical cerclage on all IVF twins in the early second trimester, so cerclage had been done on them. And in other IVF centers where cerclage was not a protocol, it was not done and taken as control. Our hospital followed the standardized transvaginal McDonald’s technique for cervical cerclage [16]. In brief, under general anesthesia, Mersilene tape of 5 mm was placed in a purse-string fashion in four passes circumferentially around the cervix, and three to four knots were placed with the ends left long enough to facilitate their removal. All patients received antibiotic prophylaxis pre-operatively but not post-procedurally. Any routine tocolytic therapy was also not advised. Patients were discharged 24-72 hours after the procedure. The cerclage was removed in the 37th gestational week, or when performing a cesarean section. Earlier removal of the cerclage was done in cases of inevitable miscarriage, premature contractions, or premature rupture of membranes.

The data was collected regarding the total number of deliveries and twins from labor room registers. There was a total of 21865 deliveries during our study period, of which 54 were IVF twins. The data of these 54 patients was collected from the medical record department using a pre-structured proforma, including parameters such as age, mode of delivery, gestational age at delivery, maternal outcome, and neonatal weight. The data collectors were blinded to the purpose of the study to overcome reviewer bias. Four monochorionic twins were excluded. Then we took out the admission medical record papers of the twins with IVF at the time of Macdonald’s cerclage. We have noted from that record that out of 54 patients, 35 had cerclage, of which 11 had ultrasound-indicated cerclage, 7 had uterine anomalies, and 2 had history-indicated cerclage were excluded from the study. Hence, a total of 30 patients - 15 twin IVF patients with prophylactic cerclage and 15 without cerclage were included in the study.

Completed weeks of gestation are considered gestation age. We have grouped participants by age at delivery as <28 weeks as extremely preterm, 28-32 weeks as very preterm, and 32-37 weeks as moderately to late preterm [17].

The primary outcome measure was gestational age at delivery of cases and control. The secondary outcome was the mode of delivery, maternal outcome, and neonatal outcome.

Definition

History-Indicated Cerclage

A history-indicated cerclage is recommended for women who have had three or more preterm deliveries and/or mid-trimester losses [6].

Ultrasound-Indicated Cerclage

An ultrasound-indicated cerclage is recommended for women with a cervical length <25 mm if they have had one or more spontaneous preterm births and/or mid-trimester losses [6].

Statistical analysis

Statistical software Jamovi 2.3.28 solid version was used for analysis of data. Continuous data was presented as mean ± SD and categorical data as percentage. To compare between means, an independent t-test was used for the continuous variable (age of participants, birth weight). Fisher exact test for categorical variables (parity, gestation age at delivery, mode of delivery, maternal outcome) as >20% of expected cell counts were less than 5. A p-value <0.05 was considered to be statistically significant.

## Results

In the total of 30 IVF twins, 15 IVF twins with cerclage were taken as cases and 15 IVF twins without cerclage as controls. The mean age of participants was 35.27 ± 5.98 years (min: 23; max: 45) in cases and 32.40 ± 5.54 years (min: 25; max: 44) in controls (Table 1).

**Table 1:** Mean age of participants in cases and controls

Mean age	Age (years) (mean ± standard deviation)
Cases (N = 15)	35.27 ± 5.98
Control (N = 15)	32.40 ± 5.54
p-value (unpaired t-test)	0.18

The parity of participants is in Table 2.

**Table 2:** Parity of participants in cases and controls

Parity (N)	Primipara	Multipara
Cases (N = 15) (% total)	5 (33.3%)	10 (66.7%)
Control (N = 15) (% total)	9 (60%)	6 (40%)
p-value (Fisher's Exact test)	0.136	

The mean gestational age at delivery of cases was 34 weeks 2 days ± 3.28 (min: 31; max: 37), and that of control was 33 weeks 5 days ± 1.66 (min: 25; max: 37). Table 3 demonstrates gestational age at delivery of participants. In cases and control groups, the births at gestational age 32-37 weeks were in maximum proportion (73.3% and 86.6%), not statistically significant (p = 1.000).

**Table 3:** Gestational age at delivery of participants in cases and controls

Gestational age at delivery (weeks)	<28	28-32	32-37
Cases (N = 15) (% total)	1 (6.7%)	3 (20.0%)	11 (73.3%)
Control (N = 15) (% total)	1 (6.7%)	1 (6.7%)	13 (86.6%)
p-value (Fisher's Exact test)	1.00		

There was no statistically significant difference between cases and controls with a p-value greater than 0.05 in the age of the participants, parity, or gestational age at delivery (Tables 1-3).

The proportion of elective lower segment cesarian section (LSCS), emergency LSCS, and preterm vaginal was 60%: 33.3%: 6.7% in control, while that in control was 13.3%: 86.7%: 0.0% which is statistically significant (p = 0.008) (Table 4).

**Table 4:** Mode of delivery of participants in cases and controls

Mode of delivery	Vaginal delivery	Elective Lower segment cesarean section	Emergency Lower segment cesarean section
Cases (N = 15) (% total)	0	2 (13.3%)	13 (86.7%)
Control (N = 15) (% total)	1 (6.7%)	9 (60.0%)	5 (33.3%)
p-value (Fisher's Exact test)	0.008		

The maternal outcome in cases and control is represented in Table 5.

**Table 5:** Maternal outcome in cases and controls

Maternal outcome	Preterm labour	Preterm rupture of membrane	Cesarean section on maternal request	Pre-eclampsia	Antepartum hemorrhage	Fetal bradycardia	Decrease fetal movement	Cesarean section for previous cesarean
Cases (N = 15) (% total)	2 (6.67%)	4 (13.34%)	2 (6.67%)	1 (3.34%)	2 (6.67%)	0	2 (6.67%)	1 (3.34%)
Control (N = 15)(% total)	1 (3.34%)	0	6 (20%)	2 (6.67%)	1 (3.34%)	4 (13.34%)	1 (3.34%)	0
p-value (Fisher's Exact test)	0.082							

The fetal outcome in terms of neonatal birth weight is tabulated in Table 6.

**Table 6:** Mean birth weight of neonates in cases and controls

Fetal outcome	Mean birth weight (gm) (mean ± standard deviation)
Cases (N = 15)	1961.33 ± 340.27
Control (N = 15)	1899.33 ± 437.48
p-value (unpaired t-test)	0.668

## Discussion

Twins with IVF is a specialized group. There is a recent study done by Mamas et al. in 2020, they have done a study over 14 years and found a total of 43 neonates with twin pregnancies with intracytoplasmic sperm injection (ICSI) IVF [9]. In our center, because of the high delivery load, we have had 30 patients in four years of deliveries. In our study, 2 (6.66%), 4 (13%), and 26 (86%) were the number of IVF twins delivered at gestational ages of <28 weeks, 28-32 weeks, and 32-37 weeks, respectively. This finding is coherent with the 2018 USA-based study on ART surveillance, which reported that gestation age of delivery at <37 weeks and <32 weeks was 61% and 10.5%, respectively [18], and the European IVF Monitoring Consortium reported preterm birth in IVF twins of 4.1% and 15.5% in 20-27 weeks and 28-32 weeks, respectively [3].

In our retrospective comparative study, no significant difference was found in terms of gestational age at delivery. The p-value of gestational age less than 38 weeks for both groups is insignificant (p = 1.000). So, prophylactic suturing did not have any positive effect on prolonging the gestational age in twin pregnancies. Previous studies showed prophylactic cerclage effective in pregnancy prolongation and minimizing preterm labor [7,8]. Another recent study done by Mamas et al. in 2020 on IVF twins showed that prophylactic cerclage has a positive effect on pregnancy outcomes, but the Shirodkar cervical cerclage technique was used in it [9].

Talking about sociodemographic features, in the study of Mamas et al. in 2020, the mean age of IVF twin presentation was 34.16 ± 3.96 years and the mean neonatal delivery weight was 2,238 ± 329 grams [9]. These were similar to our study with a mean age of participants of 35.27 ± 5.98 years in cases and 32.40 ± 5.54 years in control and birth weight of 1961.33 ± 340 gram and 1899.33 ± 437.48 grams, respectively. This shows that IVF twin women present at a late age and have less neonatal birth weight either because of preterm delivery or growth restriction.

Regarding mode of delivery, in our study, it became significant as one control delivered preterm at 25 weeks, in the rest other LSCS were mode of delivery. There is a higher incidence of LSCS in twin IVF [19].

It may be argued that IVF with single embryo transfer (SET) will avoid multiple pregnancies. However, this is applicable to women <36 years old; women usually present after 36 years for IVF [20]. It has also been reported that even with SET, the chance of twin-by-zygotic splitting is 1.56%, which is still higher than spontaneously conceived twin pregnancies [21]. Moreover, IVF patients have a powerful desire to become pregnant; they welcome multiple pregnancies, even if there are associated risks [22]. So, we cannot prevent twins in IVF, but we have to find out measures to prevent preterm delivery in IVF twins. Studying on a large scale is required for that.

Strengths and limitations

The retrospective design of this study raises the possibility of biases. The fact that the study was conducted in a single institution with a very small number of patients may limit the statistical power to detect small but clinically relevant differences and the generalizability of the results to different populations. On the other hand, the main strengths of our study include the relatively homogeneous cohort of patients and the use of a standardized preoperative protocol and surgical technique.

## Conclusions

Prophylactic transvaginal cerclage is not effective in twin IVF pregnancies with normal cervical length for preventing preterm births. There is no statistically significant difference in gestational age at delivery with or without transvaginal cervical cerclage. They mainly deliver at or near term; either cerclage is done or not. IVF twin women usually present at a late age, and their birth weight is also low. SET is not a solution to prevent twin pregnancy and, therefore, preterm birth. We have to find other methods that can prevent preterm birth in twin pregnancies. Further studies are needed on a large scale to find out preventive measures.
